# Killing two birds with one stone: emergence of colistin and cefiderocol resistance in a mucoid MDR *Acinetobacter baumannii* under colistin pressure

**DOI:** 10.3389/fmicb.2025.1650028

**Published:** 2025-09-30

**Authors:** Valeria Fox, Gianluca Vrenna, Martina Rossitto, Irene Paris, Rosanna Papa, Marco Artini, Laura Selan, Serena Raimondi, Vanessa Tuccio Guarna Assanti, Nour Essa, Maria Stefania Lepanto, Venere Cortazzo, Marilena Agosta, Alessandra Boni, Luca Cristiani, Renato Cutrera, Carlo Federico Perno, Paola Bernaschi

**Affiliations:** ^1^Multimodal Laboratory Medicine, Bambino Gesù Children’s Hospital, IRCCS, Rome, Italy; ^2^Department of Public Health and Infectious Diseases, Sapienza University, Rome, Italy; ^3^Microbiology and Diagnostic Immunology Unit, Bambino Gesù Children's Hospital, IRCCS, Rome, Italy; ^4^Pneumology and Cystic Fibrosis Unit, Bambino Gesù Children Hospital, IRCCS, Rome, Italy

**Keywords:** *Acinetobacter baumannii*, multidrug-resistant, colistin, cefiderocol, hypermucoid, resistance

## Abstract

*Acinetobacter baumannii* is a multidrug-resistant (MDR) pathogen associated with nosocomial infections, sporadically detected in cystic fibrosis (CF) patients. Treatment of *A. baumannii* may be hindered by polysaccharide capsule production of some isolates and extended resistance to most antibiotics. In these fearsome cases, colistin (COL) and cefiderocol (FDC) are considered last resort antibiotics. Unfortunately, resistance to these molecules is increasing. Indeed, we observed a hypermucoid (HM) *A. baumannii* strain producing OXA-23, isolated from a CF patient, rapidly evolving concomitant resistance to COL and FDC. At her first visit to our hospital, the 24-year-old female with a delayed CF diagnosis and advanced lung disease presented with one HM and one low mucoid (LM) *A. baumannii* phenotypes. Due to *Pseudomonas aeruginosa* infection, she received inhaled tobramycin and COL treatment. Five months later, two HM strains were isolated, with different susceptibility profiles to COL and FDC, one being completely resistant. Whole genome sequencing revealed that all four isolates, the initial HM and LM strains and the two subsequent HM strains, belonged to Sequence Type 2 and carried OXA-23 gene. Genetic distance revealed evolution from the same strain. HM strains carried mutations in genes involved in polysaccharide production while the resistant strain also harboured mutations conferring COL and FDC resistance. Biofilm production and motility of the four strains were evaluated to establish possible links between multiresistance, mucoidity and virulence. Phenotypic characterisation showed that HM strains lost some virulence traits during chronicisation and resistance development but likely persisted by exploiting the biofilm-mediated protection, maintaining both virulent and resistant subpopulations. We speculate that COL treatment forced *A. baumannii* resistance occurrence in a bacterial population already heteroresistant to FDC, resulting in a pan-resistant strain in this CF patient. Considering that lung transplantation still represents a life-saving option for CF patient with advanced lung disease, this study highlights the critical need for careful administration of last-resort molecules in patients that may face immunosuppression. Indeed, given the possibility of simultaneous emergence of resistance and the limited treatment options available to patients infected with MDR *A. baumannii,* last-resort antibiotics should be spared to avoid selection of pan-resistant microorganisms.

## 1 Introduction

*Acinetobacter baumannii* (*A. baumannii*) is universally recognised as one of the multidrug-resistant (MDR) pathogens most associated with nosocomial infections (Nowak et al., 2017). Due to its ability to rapidly develop resistance, by acquiring or upregulating various resistance determinants, *A. baumannii* poses a potential threat to human health and it is considered a top priority pathogen for which a comprehensive assessment of pathogenic mechanisms is urgently needed (Kurihara et al., 2020). Furthermore, the propensity of *A. baumannii* to adhere and persist on surfaces in biofilm phenotype has contributed to its pathogenicity and drug resistance, facilitating its survival in hospital environment (Nowak et al., 2017; Artini et al., 2025). Several nosocomial infections are increasingly being caused by extensively drug-resistant (XDR) and pandrug-resistant (PDR) isolates of *A. baumannii,* particularly in southern Europe, affecting primarily vulnerable patient groups such as intensive care unit (ICU) patients (Nowak et al., 2017). Mortality rates for patients with *A. baumannii* healthcare-associated infections vary according to local epidemiology and diffusion of resistant phenotypes and carbapenem-resistant *A. baumannii* (CRAB), ranging from 29% to more than 90% in ICU patients (Corcione et al., 2024). Indeed, several mechanisms can drive *A. baumannii* multi-drug resistance: antibiotics sensitivity can be affected by non-enzymatic mechanisms such as efflux pumps (e.g., aminoglycosides, chloramphenicol, glycopeptides), reduced membrane permeability (e.g., carbapenem or aztreonam) and target site modifications (e.g., fluoroquinolones, rifampicin or colistin) (Vrancianu et al., 2020). Furthermore, enzymatic mechanisms, particularly the production of *β*-lactamase, are often involved in antibiotic resistance, especially against carbapenems: indeed *A. baumannii* can harbour all the four Ambler β-lactamase classes, with class D oxacillinases (OXA) such as OXA-23, either plasmid-borne or chromosomally encoded, being the most widespread worldwide (Vrancianu et al., 2020; Hamidian and Nigro, 2019). Carbapenems have long been used as last-resort antibiotics for the treatment of infections caused by MDR *A. baumannii*, however CRAB nosocomial outbreaks have reached worldwide diffusion, causing widespread clinical concerns (Hamidian and Nigro, 2019). Therefore, for difficult-to-treat infections, alternative molecules are under evaluation (e.g., rifabutin and zosurabalpin) while others are employed as last-resort therapies, such as sulbactam/durlobactam, colistin (COL) and cefiderocol (FDC); however, resistance to these drugs is increasingly being reported (Bostanghadiri et al., 2024; Zhan et al., 2024; Dubey et al., 2025). In addition, complicating antibiotic treatment and contributing to chronicity and colonisation, mucoid-type isolates of *A. baumannii* have recently been identified, with a polysaccharide capsule involved in biofilm formation, that hampers eradication possibility (Shan et al., 2021). While the mucoid phenotype is a factor in hypervirulence in other Gram-negative and Gram-positive bacteria, links between the ability to produce biofilms, multi-resistance, motility and mucoidy in *A. baumannii* are still unclear (Shan et al., 2021).

We recently described the first isolation of a mucoid strain of *A. baumannii* producing OXA-23 in a patient with cystic fibrosis (CF) (Rossitto et al., 2023), a genetic disease that involves chronic bacterial colonization of the lungs, causing lung deterioration, and eventually requiring lung transplantation as a last-resort, life-saving option. Except for occasional findings, *A. baumannii* producing OXA-23 is not a common coloniser of CF patients’ lung; however, since the patient had been previously admitted to ICU, a well-known niche for *A. baumannii,* we hypothesised that this rare infection became established on that occasion (Rossitto et al., 2023). Notably, the patient appeared to be colonised by two different phenotypes, one being characterised by an exceptionally long viscous strings of 150 mm, classifying as an hypermucoid morphotype (Gong et al., 2022).

Probably, as suggested by the preexisting colonisation from a mucoid phenotype of *P. aeruginosa,* patient’s compromised lung capacity, together with the stressful environment typical of CF lung, may have driven evolution toward such hypermucoid phenotype. In the follow-up of this same patient, colonizing *A. baumannii* showed unexpected and undesirable adaptation to the antibiotic regimen to which the patient had been subjected, showing rapid emergence of concomitant resistance to COL and FDC.

Here we report the genomic and phenotypic characterisation of the *A. baumannii* strains isolated before and after resistance evolution, investigating the causes of modifications in antimicrobial susceptibility and virulence traits.

## 2 Materials and methods

### 2.1 Bacterial isolates

During the patient’s first visit to our hospital, 3 months after an ICU hospitalization for a right upper lobe segmentectomy, two phenotypes of *A. baumannii* were isolated from a bronchoalveolar lavage along with mucoid *Pseudomonas aeruginosa*: a hypermucoid (HM) phenotype named AM3, with an opaque appearance, and a low mucoid (LM) phenotype named AM4, with a translucent appearance.

Two additional HM *A. baumannii* strains, AM61 and AM62, were isolated at a follow-up visit five months later, after continuous and alternating inhaled antibiotic therapy with tobramycin and COL for maintenance therapy of the *P. aeruginosa* infection.

The patient provided consent to use personal data for diagnosis, treatment and related future research purposes at the time of hospitalization.

### 2.2 Susceptibility test and whole genome sequencing (WGS)

Frozen bacterial stocks were plated on Columbia agar + 5% sheep blood (bioMérieux, Marcy l’Etoile, France) incubated overnight at 37 °C. Susceptibility testing was performed by the broth microdilution method using the MicroScan panel NMDR2 (Beckman Coulter, Indianapolis, IN, United States) and were interpreted according to clinical breakpoints based on the European Committee on Antimicrobial Susceptibility Testing (EUCAST) tables. The manufacturer’s procedures were followed for testing ComASP Cefiderocol (Liofilchem, Roseto degli Abruzzi, Italy). MIC reference values were determined using iron-depleted CA-MHB, prepared following EUCAST guidelines, with FDC concentrations ranging from 0.016 to 16 mg/L. The readings were conducted by two operators, with a third operator verifying results for plates that were difficult to interpret. Panels were inoculated using a single- or multichannel pipette, sealed with the provided seal, and incubated at 36 ± 2 °C for 16–20 h in ambient air. The panels were read manually, using bright indirect lighting against a dark background to enhance readability if needed.

Bacterial DNA was extracted using the EZ1 extractor (Qiagen BioRobot EZ1, Qiagen, Hilden, Germany) using the proper extraction kit (EZ1&2 DNA tissue kit, Qiagen, Hilden, Germany), following the manufacturer’s instructions. Next Generation Sequencing library preparation was performed according to the manufacturer’s protocol with the DNAprep kit (Illumina, San Diego, CA, United States). Prepared libraries were sequenced using MiSeq Reagent Kit v3 to obtain 2×150 bp paired-end reads, and sequenced on a MiSeq instrument (Illumina, San Diego, CA, United States). Bioinformatic analysis was performed to identify bacterial Sequence Type (ST), Single Nucleotide Polymorphisms (SNPs), virulence and resistance genes. The raw reads were pre-processed with Fastp (v0.23.4) (Chen et al., 2018), filtering for adapters and quality (Phred score > 28) and then quality checked with FastQC (v0.11.9) (Andrews, 2010). Taxonomic classification was performed with Kraken2 (v2.1.3) (Wood et al., 2019) to screen for potential contaminations. Genome assembly was performed *de novo* with Shovill (v1.1.0) (Seemann, 2017), checking the assembly quality with Quast (v5.1) (Gurevich et al., 2013) and subsequently annotating with Prokka (v1.14.6) (Seemann, 2014). Multilocus Sequence Type (MLST) was assessed with MLST tool (v2.11) (Seemann, 2024a) using the Pasteur and Oxford schemes. Investigation of antibiotic resistance and virulence determinants was performed using ABRicate (v0.4) (Seemann, 2024b) using the Comprehensive Antibiotic Resistance Database (CARD) (Jia et al., 2017) and Virulence Factor Database (VFDB) (Chen et al., 2016), respectively, with 90% coverage (−mincov) and 90% identity (−minid) parameters. Mutations in genes involved in different virulence mechanisms (Biofilm, Type IV pili, Immune modulation, and Effector delivery systems) were assessed by mapping reads with bwa mem (Li, 2013) against the *A. baumannii* ACICU reference strain (GenBank accession number CP000863.1). Phylogenetic analysis was performed by performing Single Nucleotide Polymorphism (SNP) calling with Snippy (v4.6.0) (Seemann, 2015), using the AB36-VUB strain (GenBank accession n° CP091371) as reference, and incorporating other *A. baumannii* genomes belonging to the same ST (Supplementary Table 1). Maximum Likelihood phylogenetic analysis was performed on a coreSNP of 9,094 bp with IqTree (v1.6.12) (Nguyen et al., 2015) using the best-fit model of nucleotide substitution TVMe+ASC + R2 with 1,000 replicates fast bootstrapping. Mobile genetic element (MGE) identification and plasmid reconstruction were carried out using the MOB-suite tool (v3.1.8) (Robertson and Nash, 2018). Differences in the core genome among the 4 strains were assessed by performing SNP calling with Snippy (v4.6.0) (Seemann, 2015), using the LM AM4 strain as reference.

### 2.3 Biofilm formation

The biofilm quantification was assessed by microtiter plate (MTP) biofilm assay (Artini et al., 2022). An overnight bacterial culture was 1:100 diluted into Brain Heart Infusion broth (BHI, Oxoid, Basingstoke, UK) medium and aliquoted in the wells of a sterile 96-well polystyrene flat base plate. The plates were incubated overnight at 37 °C under static conditions in aerophilic and microaerophilic (5% CO_2_) conditions for 24 h. Notably, microaerophilic conditions were included to better reproduce the atmospheric environment of the cystic fibrosis lung. After incubation, the supernatant containing planktonic cells were gently removed from the multiwells, and the plates were washed with double-distilled water. Then the microtiter plates were patted dry in an inverted position. The staining was performed with 0.5% crystal violet for 15 min at room temperature. The excess of crystal violet was removed by washing the wells with double-distilled water. The microtiter plates were thoroughly dried. The remaining biofilm was dissolved with 20% (*v*/*v*) glacial acetic acid and 80% (*v*/*v*) ethanol, for 20 min under agitation at room temperature. The biofilm content was spectrophotometrically measured at 590 nm. For the determination of biofilm formation at 48 h, after 24 h of growth, the supernatant containing planktonic cells was sterile removed and replaced with 100 μL of fresh BHI medium. These multiwells were incubated at 37 °C for an additional 24 h in aerophilic and microaerophilic conditions. At the end of the incubation, biofilm was quantified through crystal violet staining as previously described.

Each experiment was performed in 6-replicates, and each data point was composed of three independent experiments.

### 2.4 Motility assays: twitching and surface motility

A single colony of *A. baumannii* was inoculated in 5 mL of Nutrient Broth (Oxoid, Basingstoke, UK) in a sterile conical bottom tube and incubated overnight at 37 °C under constant stirring at 180 rpm. The semisolid medium used for twitching and surface-associated motilities was prepared with 0.5% Tryptone (Oxoid, Basingstoke, UK), 0.25% sodium chloride (Sigma, Steinheim, Germany), and 0.3% agarose (Invitrogen, Paisley, UK). After autoclaving, the medium was deposited into 6-well polystyrene plates and allowed to solidify. For the study of twitching motility, 2 μL of overnight bacterial culture were inoculated on the bottom of the well (between the semisolid medium and the plastic) while, for investigating surface motility, 2 μL of overnight inoculum were inoculated on the surface of the semisolid medium. Subsequently, multiwells were incubated at 37 °C both in aerophilic and microaerophilic conditions. Motility was analyzed at 24, 48 and 72 h.

The surface-associated motility was analysed on the surface of a semi-solid medium (medium/air interface), while twitching motility was analysed for bacteria moving between the bottom of the polystyrene plate and the semi-solid medium. The study of the two different motilities was conducted simultaneously using the same well and medium. Furthermore, both motilities were observed after incubating the plates at 37 °C for 24, 48 and 72 h in normoxia (aerophilic condition) and in an atmosphere with 5% CO_2_ (microaerophilic condition).

Twitching motility was observed by placing the plates against the light, it was possible to observe a halo produced by bacteria moved in eccentric directions between the bottom of the wells of a sterile 6-well plate and the semi-solid medium (Corral et al., 2021).

### 2.5 Statistical analysis of biological evaluation

Data reported were statistically validated using Student’s t-test comparing mean diameters of motility in the different experimental conditions. The significance of differences was calculated using a two-tailed Student’s t-test. A *p* value of <0.05 was considered significant.

## 3 Results

**Figure 1:**
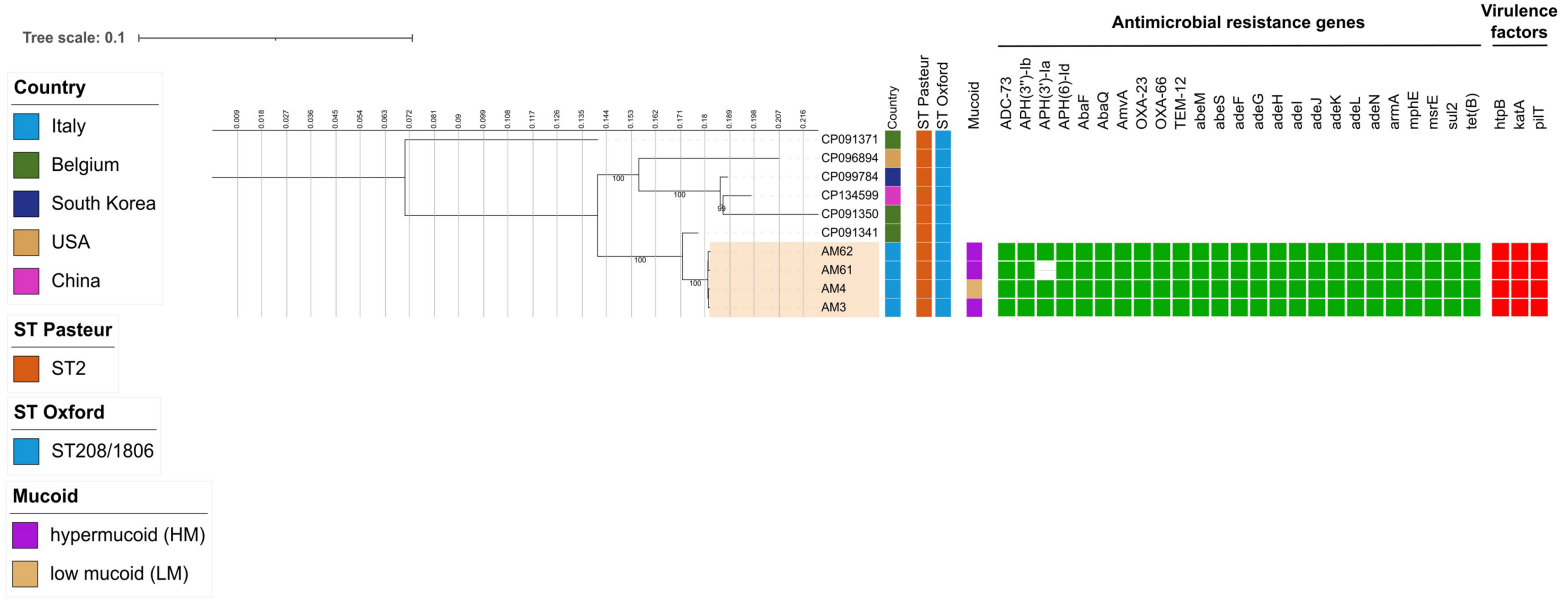
Estimated maximum likelihood phylogenetic analysis of *A. baumannii* isolates (*n* = 4) and reference genomes belonging to the same ST (*n* = 6). The phylogeny was estimated on a coreSNP of 2,282 bp with IqTree using the best-fit model of nucleotide substitution TVMe + ASC with 1,000 replicates fast bootstrapping. Leaves number represents the sample IDs, bootstraps values higher than 90 are shown on branches. Information regarding the samples is shown: Sequence Type following the Pasteur and Oxford schemes (ST Pasteur and ST Oxford), presence of hyper/low mucoid phenotype (Mucoid), presence (filled green square) or absence of antimicrobial resistance genes (AMR), presence (filled red square) or absence of virulence factors.

Both HM *A. baumannii* (AM3) and LM *A. baumannii* (AM4) strains deriving from the first sample showed MDR profile, but low Minimum Inhibitory Concentration (MIC) to COL and FDC (Table 1). In the second sample, translucent *A. baumannii* was no longer detectable, while HM *A. baumannii* had two different susceptibility profiles to COL and FDC, one with a low MIC (AM61) and the other one (AM62) with a MIC above the upper tested value (Table 1). No other changes in MICs were detected compared to the previous isolated strain (Supplementary Table 2). WGS analysis revealed that all four *A. baumannii* isolates belonged to the Pasteur Sequence Type (ST2) and Oxford ST208/1806 (Figure 1). At the phylogenetic analysis, they resulted to be close to a clinical strain isolated in Belgium in 2017 from endotracheal aspirates (Figure 1), reported as extensively drug-resistant on Acinetobase (n.d.). Regarding genetic features related to virulence and antimicrobial resistance, all four shared the same genes, with the exception of AM61 strain, which lacked the APH(3′)-Ia gene (Figure 1). By plasmid reconstruction, all four strains were found to carry the OXA-23 gene located on a plasmid homologous to *A. baumannii* plasmid pORAB01-2 (GenBank accession number CP015485). A list of all plasmids and mobile genetic elements (MGEs) found is available in the Supplementary Table 3.

**Table 1:** Susceptibility to colistin and cefiderocol of the four *A. baumannii* strains.

Strain ID	*A. baumannii* phenotype	Isolation date	COL MIC (mg/L)	FDC MIC (mg/L)
AM3	HM	04/12/2022	≤ 2	≤ 2
AM4	LM	04/12/2022	≤ 2	≤ 2
AM61	HM	10/05/2023	≤ 2	≤ 2
AM62	HM	10/05/2023	≥ 16	≥ 128

HM, hypermucoid; LM, low mucoid; COL, Colistin; MIC, Minimum Inhibitory Concentration; FDC, Cefiderocol.

In terms of genetic distance, it was observed that they can be considered as an evolution of the same strain, as they differed for a median (IQR) distance of 12 (Dubey et al., 2025; Shan et al., 2021; Rossitto et al., 2023) Single Nucleotide Polymorphisms (SNPs). By looking at the differences among the 4 strains, SNPs analysis also revealed that HM *A. baumannii* strains had missense mutations in the capsular locus *wzc* (*ptk*) gene, involved in polysaccharide production, namely C1592T (resulting in A531V substitution) and G1957A (resulting in M653V substitution) in AM3 strain, and only G1957A (M653V) in AM61 and AM62 strains, respectively (the full list of SNPs is available in Supplementary Table 3). The HM *A. baumannii* strain with high MICs to COL and FDC in the second sample (AM62) had missense mutations in *pmrB* (C425T, resulting in A412V substitution) and *piuA* (A911G, resulting in D304G substitution) genes, respectively. Both mutations were already found in literature to be implicated in colistin (A142V in *pmrB*) and cefiderocol (A911G in *piuA*) resistance in *A. baumannii* (Haeili et al., 2018; Karakonstantis et al., 2022; Novović and Jovčić, 2023).

In order to establish possible links between multi-resistance, mucoidy and virulence, we evaluated biofilm production and motility for the four *A. baumannii* strains. Two different oxygen conditions were evaluated to more accurately investigate potential phenotypic differences between the lungs of CF patients, characterized by a microaerophilic environment (5% CO2), and those of non-CF individuals (aerophilic condition).

**Figure 2:**
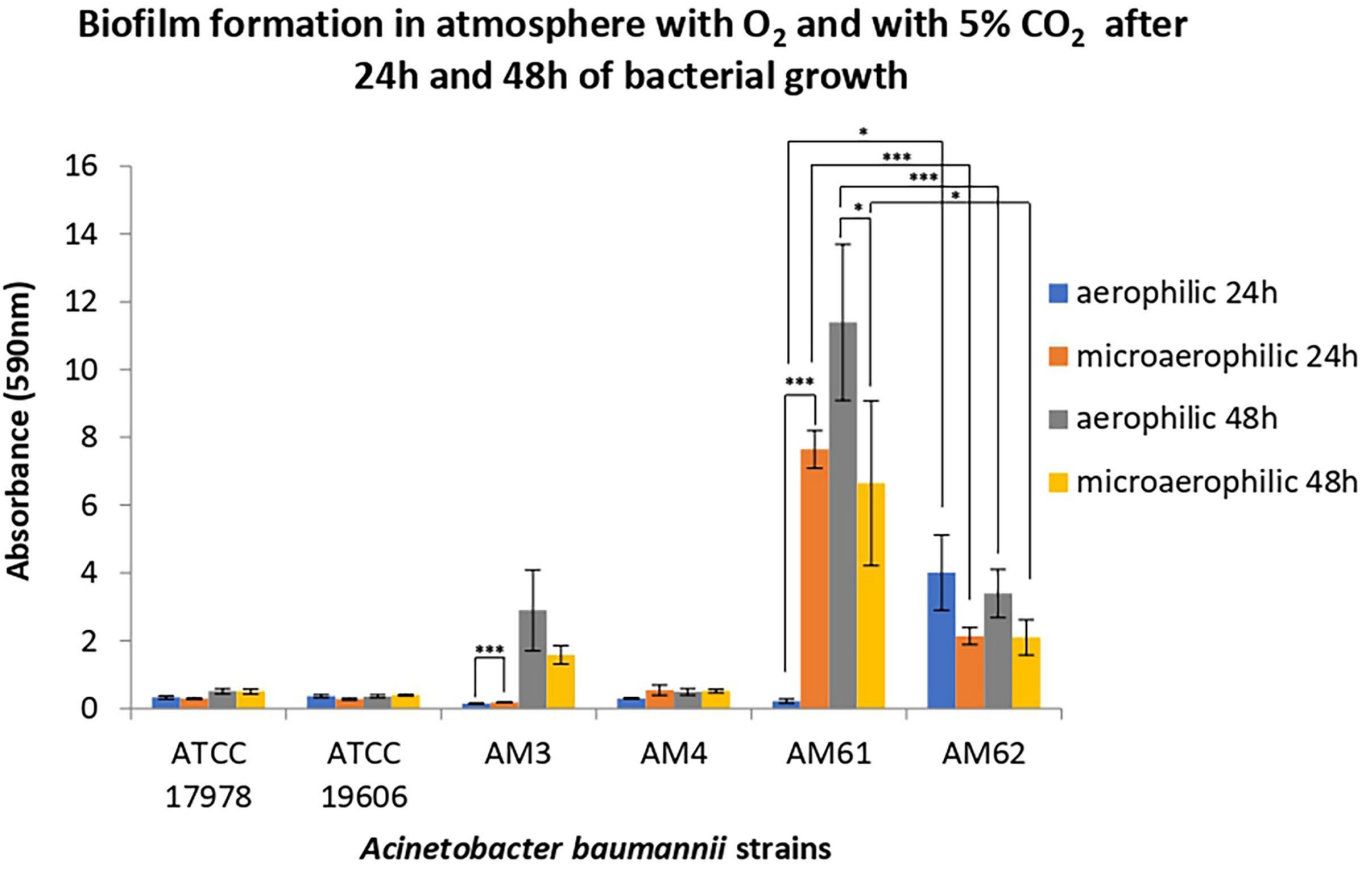
Biofilm formation of reference and clinical isolates of *A. baumannii*. Biofilm formation was evaluated after 24 and 48 h of bacterial growth in an atmosphere with O_2_ and with 5% CO_2_. Data are reported as OD at 590 nm after crystal violet staining. Each data point represents the mean ± SD of 3 independent experiments, each performed in 6-replicates. Error bars indicate the standard deviations of all the measurements. Statistical difference was determined by Student’s t-test: * *p* < 0.05; ** *p* < 0.01; *** *p* < 0,001.

Biofilm formation was assessed in aerophilic and microaerophilic conditions after 24 h and 48 h of incubation at 37 °C for all four clinical strains, compared to two reference strains (Figure 2). The reference strains ATCC 17978 and ATCC 19606, selected as low biofilm producers and non-mucoid, differ in their resistance profiles, with ATCC 17978 being antibiotic-sensitive and ATCC 19606 multidrug-resistant. As shown in Figure 2, both reference strains, despite differing in resistance profiles, exhibited no changes in biofilm production under the two oxygen conditions, consistent with the behaviour of the clinical LM AM4 strain.

Overall, while the LM AM4 strain did not form biofilm under any incubation conditions, mucoid strains from both samples did form biofilm, with different capabilities depending by incubation conditions. In particular, the first HM strain isolated, AM3, produced biofilm only after 48 h of incubation, showing a slight increase under aerobic conditions compared to microaerophilic conditions. Between the strains isolated from the second sample, the “sensitive” HM strain AM61 showed highest total biofilm production, peaking after 48 h of incubation under aerobic conditions. Conversely, the ‘resistant’ HM strain AM62 produced significantly less biofilm than AM61, maintaining the levels of the ‘ancestral’ AM3 in all but one condition. Indeed, AM62 seemed to be the only strain capable of producing biomass after 24 h in aerobiosis. Finally, both AM3 and AM61 produced significantly more biofilm in microaerophilic conditions compared to aerophilic conditions after 24 h. This observation was reversed for the “resistant” HM strain AM62, which reduced biomass moving from aerophilia to microaerophilia after 24 h.

**Figure 3:**
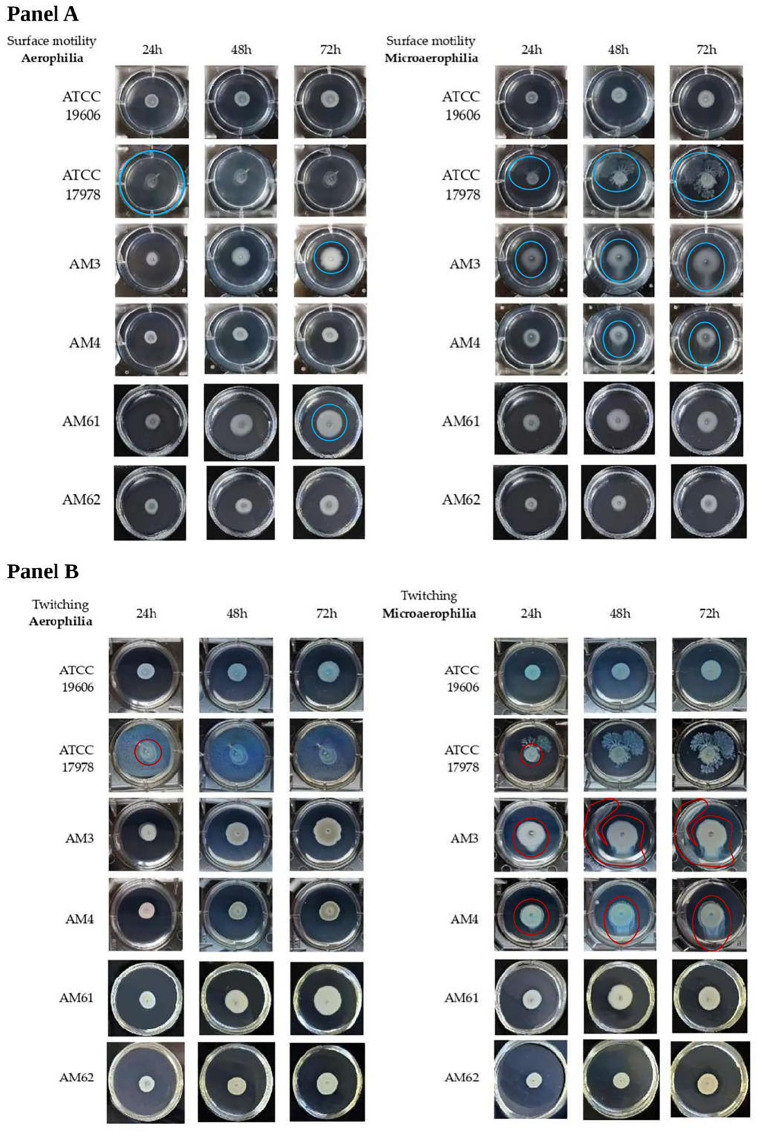
Motility of *A. baumannii* bacterial strains in different oxygen conditions. **(A)** Surface-associated motility of *A. baumannii* bacterial strains in aerophilic (left panel) and microerophilic (right panel) conditions of bacterial growth. The motility halo was highlighted with a blue circle. The reference strain ATCC 17978 was used as a positive control for this motility, producing a motility halo of 3.5 cm (covering the entire well) within just 24 h under aerobic growth conditions. Instead, under microaerophilic conditions, this strain displayed a branched motility pattern, with the halo diameter gradually increasing over time. Conversely, the ATCC 19606 strain, used as a negative control, did not exhibit this motility in either of the growth conditions tested. **(B)** Twitching motility of *A. baumannii* bacterial strains in aerophilic (left panel) and microerophilic (right panel) conditions of bacterial growth. Twitching motility was visualized by backlighting the multiwell plates and is highlighted with red circles. ATCC 17978 and ATCC 19606 reference strains did not exhibit twitching motility under tested conditions. For ATCC 17978 strain, the halo highlighted with a red circle corresponded to the initial drop placed on the bottom of the plate. The two different motilities were analyzed at 24, 48 and 72 h.

Surface-associated (Figure 3A) and twitching motilities (Figure 3B) of *A. baumannii* strains were analysed at 24, 48 and 72 h, in both aerophilic and microaerophilic conditions. Overall, surface-associated motility appeared to be a characteristic of the HM strains, except for the “resistant” HM, while twitching motility appeared to be a characteristic of the only strains isolated from the first sample.

Although *A. baumannii* has long been defined as non-motile, it has been demonstrated that it possesses two kinds of motilities defined as surface-associated and twitching motility (Jeong et al., 2024). Surface-associated motility was observed as a bacterial halo on the medium-air interface, whereas twitching motility was assessed by bacterial movement between the bottom of the polystyrene plate and the medium. Both motility assays were performed simultaneously in the same well using soft agar medium.

**Figure 4:**
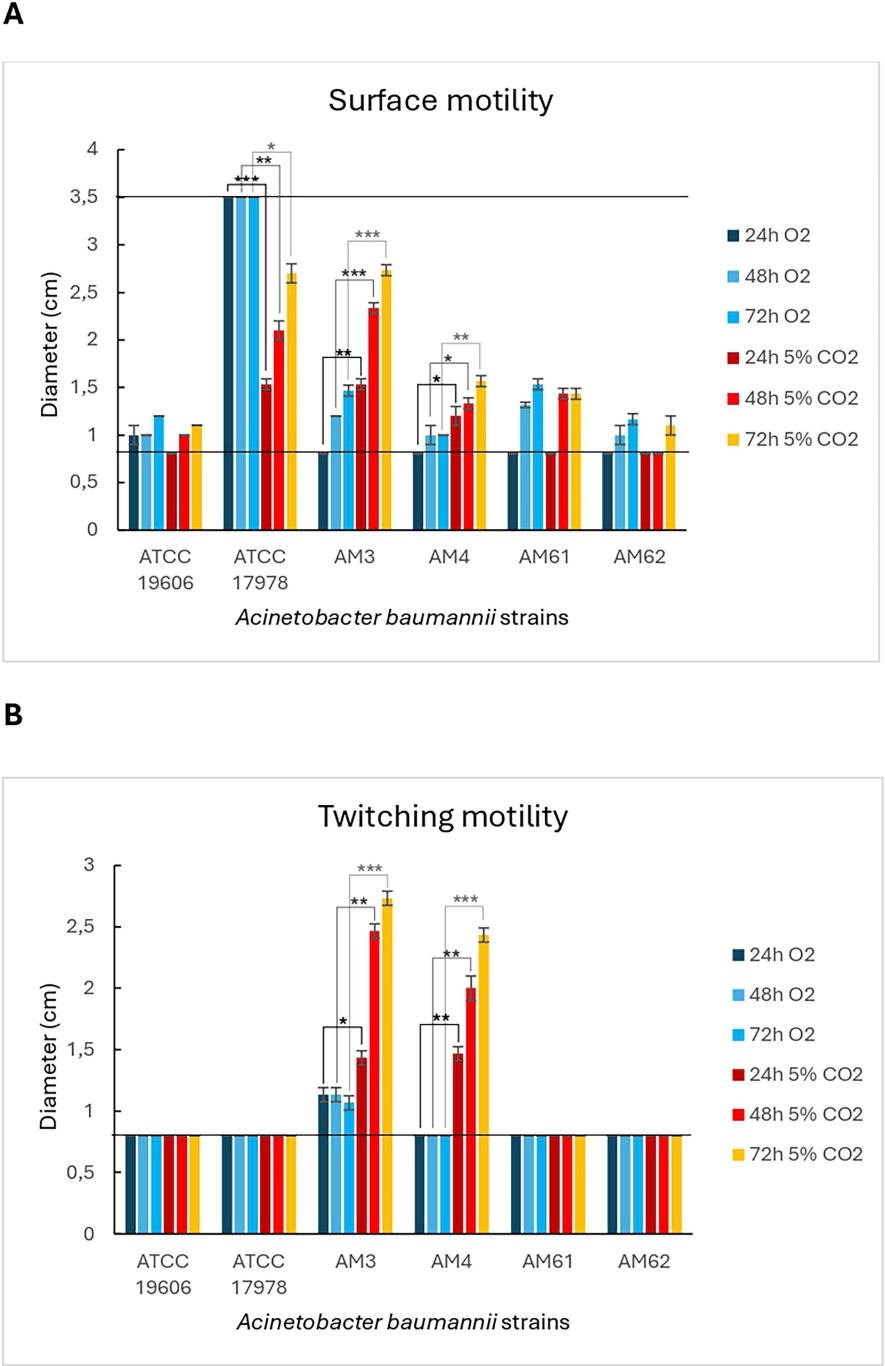
Diameters of motility of *A. baumannii* bacterial strains in different oxygen conditions. **(A)** Surface-associated motility. Bar-graph showing the diameters of the surface motility halos for the different strains tested under aerobic and microaerophilic growth conditions. The lowest (0.8 cm) and highest (3.5 cm) diameters are indicated by black lines, corresponding to the initial drop and the well size, respectively. **(B)** Twitching motility. Bar-graph showing the diameters of the twitching motility halos for the different strains tested under aerobic and microaerophilic growth conditions. The lowest (0.8 cm) diameter is indicated by a black line, corresponding to the initial drop size. Statistical analysis was determined with Student’s T-test (* *p* < 0.05; ** *p* < 0.01; *** *p* < 0.001).

In Figure 3A, the surface motility halo was highlighted with a blue circle, while Figure 4A showed the measured halo diameters. The maximum diameter was 3.5 cm (well size), while the minimum diameter was 0.8 cm (initial drop). The reference strain ATCC 17978 was used as a positive control for this motility, producing a motility halo of 3.5 cm (covering the entire well) within just 24 h under aerobic growth conditions. Instead, under microaerophilic conditions, this strain displayed a branched motility pattern, with the halo diameter gradually increasing over time. Conversely, the ATCC 19606 strain, used as a negative control, did not exhibit this motility in either of the growth conditions tested, as evidenced in Figures 3A, 4A. Among the clinical isolates, surface-associated motility was exhibited only by HM AM3 and the “sensitive” HM AM61 strain. While AM3 from the first sample seemed to prefer microaerophilic conditions over aerobiosis, AM61 showed the opposite behaviour. In fact, AM3 showed consistent motility already after 24 h in microaerophilia (right panel of Figure 3A) and only after 72 h in aerobiosis (left panel of Figure 3A), whereas AM61 showed less surface-associated motility moving from aerobiosis to microaerophilia. The differences highlighted for each strain in the different oxygen conditions resulted to be significative for ATCC17978 and AM3 and AM4.

Twitching motility, located below the semi-solid medium, was visualized by backlighting the multiwell plates and is highlighted in Figure 3B with red circles. Figure 4B showed the measured halo diameters. Neither of the reference strains exhibited this motility under any of the conditions tested. Indeed, for the ATCC 17978 strain, the halo highlighted with a red circle corresponded to the initial drop placed on the bottom of the plate.

Twitching motility was not observed in any of the strains analysed under aerobic conditions (left panel of Figure 3B). However, microaerophilia seemed to stimulate such motility in the strains from the first sample, HM AM3 and LM AM4, after 48 h of incubation (right panel of Figure 3B). Statical analysis reported showed that these differences are significative (Figure 4B).

Overall, we could not identify mutations in genes associated with biofilm and motility that would account for these differences (see Supplementary Table 3). It is likely that these differences might arise from subtle and fine regulatory changes, rather than coding sequencies alterations. Such changes could include modulation of gene expression or post-transcriptional regulation, which might regulate multifactorial processes like biofilm formation, motility, and surface adherence. These mechanisms could also allow the strains to rapidly respond and adapt to the complex environment of the CF lung, which is characterized by the presence of multiple stressors, such as immune responses, nutrient limitation, as well as antibiotic pressure, all factors that could influence bacterial behaviour without specifically requiring the emergence of mutation in virulence factors.

## 4 Discussion

Infections caused by MDR *A. baumannii* are difficult to treat, and resistance is emerging even for antibiotics that have long been considered as a last resort. In our case, the airways of a CF patient were initially colonised by two different phenotypes of MDR *A. baumannii* producing OXA-23 carbapenemase, an opaque (mucoid or hypermucoid-HM according to our definition) and a translucent (non-mucoid or low mucoid-LM) variant. As part of a standard protocol of maintenance therapy for *P. aeruginosa* colonisation, the patient was prescribed an on/off regimen with inhaled tobramycin and COL, the latter being one of the few susceptibilities conserved by both *A. baumannii* phenotypes. When the patient returned five months later, the LM *A. baumannii* phenotype had been lost under antibiotic pressure, whereas the HM *A. baumannii* persisted and adapted to COL through LPS modification by altering PmrB, part of the two-component system mainly associated with resistance to colistin *in vivo* (Marano et al., 2020). This observation is consistent with previous experimental findings showing that the opaque *A. baumannii* variant has a fitness advantage over its translucent counterpart, as opaque colonies were found to be surrounded by extracellular polysaccharide moieties rich in N-acetylglucosamine residues that seem to protect individual cells from colistin-mediated killing, and, together with mushroom-shaped biofilm structures, further shield bacterial communities from drug, allowing higher tolerance to subinhibitory concentrations of COL (Mushtaq et al., 2024). Indeed, the extracellular polysaccharide moieties and unique biofilm structures allowed the opaque *A. baumannii* to tolerate COL exposure at both single cell and community levels (Mushtaq et al., 2024). This protective effect at the community level may explain why, in our case, the HM variant could persist in the host despite prolonged colistin exposure and how a subpopulation of the HM phenotype retained its sensitivity to COL.

Unfortunately, the COL-resistant HM subpopulation also displayed FDC resistance caused by a mutation in a TonB-dependent siderophore receptor. While this mutation does not directly confer colistin resistance, its presence under colistin treatment likely reflects selection of a pre-existing subpopulation already carrying the TonB mutation. The emergence of this resistance during treatment with FDC, as well as cross-resistance induced by ceftazidime/avibactam and ceftolozane/tazobactam, has already been described, whereas cross-resistance between COL and FDC was only considered potential (Karakonstantis et al., 2022). Given the high prevalence of FDC heteroresistance (60%) in carbapenem-resistant *A. baumannii*, it is plausible that colistin treatment indirectly enriched for HM cells with pre-existing FDC resistance, rather than selecting this mutation *de novo*. Indeed, the emergence of FDC resistance in *A. baumannii* without prior FDC treatment has been reported, supporting the notion that resistant subpopulations may pre-exist and be selected under other antibiotic pressures (Kollef et al., 2023; Alteri et al., 2024). Further studies are required to determine the precise fitness effects of TonB mutations under colistin exposure.

In addition to phenotypic changes and antibiotic resistance, *A. baumannii* colony variation is also associated with differences in surface motility and biofilm formation (Mushtaq et al., 2024), characteristics highly variable in conditions mimicking the CF lung environment (i.e., microaerophilia). *A. baumannii* virulence is directly tied to motility, and previous studies have shown that the ‘opaque variant’ is characterised by improved surface-associated motility. In our case, only the opaque strains exhibited this kind of movement, likely driven by the release of additional extracellular polymeric substances, such as polysaccharides, which reduce surface friction and enable sliding motility (Jeong et al., 2024), with the exception of the resistant HM AM62. This suggests that the emergence of resistance leads to the loss of virulence factors, given the associated fitness costs. Additionally, contrary to previous findings suggesting correlation between surface-motility and biofilm formation (Blaschke et al., 2021), surface-motility did not seem to correlate strictly with biofilm production. Indeed the ‘motile’ AM3 produced approximately the same amount of biofilm as the ‘nonmotile’ AM62. Another important component of overall motility in *A. baumannii* is twitching motility, mediated by extension and retraction of type IV pili (Clemmer et al., 2011). In our case, this type of motility was only observed in the HM and LM strains from the first sample, indicating that *A. baumannii* reduced, as expected, motility during colonisation progression and chronicisation process. Thus, in this particular case, the development of resistance and progressive adaptation to CF forced *A. baumannii* to lose some of its virulence factors. However, likely by taking advantage of the protective effect exerted on a community level, the particular *A. baumannii* clone that colonised the patient persisted in both virulent and resistant forms.

The occurrence of armful persistence of such colonizing bacteria should be actively avoided, especially when it concerns patients who are at risk of becoming immunosuppressed and in need of a solid organ transplant. In this case, a different strategy for patient’s *P. aeruginosa* infection maintenance could have been implemented, given its extensive antibiotic sensitivity, thus presumably preventing the emergence of a difficult-to-treat strain of pan resistant *A. baumannii*. Instead of colistin, levofloxacin or aztreonam could have been prescribed, as these are common molecules adopted by our Centre for maintenance therapy and the *P. aeruginosa* strain was completely sensitive to them. Taken all together and considering the ominous event of simultaneous appearance of resistance to two antibiotics used as last-resort therapy, this case highlights the critical need for careful administration of such molecules, guided whenever possible by diagnostic and antimicrobial stewardship, given the limited treatment options available for patients infected with MDR *A. baumannii*.

## Data Availability

The datasets presented in this study can be found in online repositories. The names of the repository/repositories and accession number(s) can be found at: https://www.ebi.ac.uk/ena, PRJEB85264.
